# Risk prediction models for postoperative pneumonia in lung cancer patients: a systematic review and meta-analysis

**DOI:** 10.3389/fonc.2026.1967523

**Published:** 2026-10-30

**Authors:** Chuansheng Li, Yunxi Jiang, Yanmei Wang

**Affiliations:** 1 Blood Purification Department, Yantai Yuhuangding Hospital, Yantai, Shandong, China; 2 School of Nursing, Qingdao University, Qingdao, Shandong, China

**Keywords:** lung cancer, meta-analysis, postoperative pneumonia, risk prediction model, systematic review

## Abstract

**Background:**

Postoperative pneumonia (POP) is a common complication after lung cancer surgery. Numerous risk prediction models for POP have been developed. However, the quality and predictive performance of these models remain unclear.

**Methods:**

A literature search was carried out in CNKI, Wanfang Database, VIP Database, SinoMed, PubMed, Embase, the Cochrane Library, CINAHL, and Web of Science. The search covered studies on risk prediction models for postoperative pneumonia in lung cancer patients from inception to July 20, 2026. The research question was formulated using the PICOTS framework. Data extraction was performed following the Critical Appraisal and Data Extraction for Systematic Reviews of Prediction Modelling Studies (CHARMS), and risk of bias and applicability were assessed using the Prediction Model Risk of Bias Assessment Tool (PROBAST). Meta-analysis was conducted using STATA 17.0 software.

**Results:**

A total of 26 prediction models from 20 studies were included. Models were developed predominantly with logistic regression, with machine-learning approaches used in two studies. Common predictors included age, operation time, smoking, diabetes mellitus, preoperative pulmonary function, chronic obstructive pulmonary disease (COPD), and TNM stage. All studies exhibited a high risk of bias, mainly due to inadequate data sources and suboptimal reporting in the analysis domain; 16 studies also raised applicability concerns, mainly due to participant selection limited to specific lung cancer subgroups. The pooled AUC from the meta-analysis was 0.89 (95% CI: 0.87-0.91).

**Conclusion:**

Although existing postoperative pneumonia prediction models for lung cancer patients show good discriminative performance, their clinical applicability is limited by a high risk of bias, which may lead to overestimation of discrimination. Future studies should develop more robust models with larger sample, rigorous designs, and multicenter external validation.

**Systematic review registration:**

https://www.crd.york.ac.uk/prospero/, identifier CRD42024554224.

## 1 Introduction

Lung cancer is a significant global public health issue (1, 2). Annually, it accounts for 2.2 million new cases and 1.8 million deaths, making it the second most common cancer by incidence and the leading cause of cancer-related deaths worldwide (3). Surgery remains the preferred and most effective treatment for resectable lung cancer (4, 5). Postoperative pneumonia (POP) is a prevalent complication following lung cancer surgery, occurring in up to 25% of cases (2, 6). POP can considerably extend hospital stays, escalate costs, and increase perioperative mortality rates (7, 8). Its occurrence adversely affects the prognosis of cancer treatment and correlates significantly with the overall survival rates of patients. Consequently, the capability to effectively detect and prevent POP is a vital aspect of perioperative care in lung cancer surgery.

Studies indicate that appropriate preventive measures can mitigate the risk of POP (9, 10). Preventing POP requires early detection and prediction, followed by targeted interventions (11, 12). Early identification also facilitates the prompt treatment of POP as it develops (13). Currently, the Physiological and Operative Severity Score for the Enumeration of Mortality and Morbidity (POSSUM) system is used to predict postoperative complications (14). However, this tool often lacks specificity for POP and may not accurately assess the risk for specific populations.

Risk prediction models estimate the likelihood of an individual developing a particular disease or outcome based on various personal characteristics (15). In recent years, numerous studies have developed or validated risk prediction models for POP in lung cancer patients. Nevertheless, there remains a lack of a universally recognized and authoritative model recommended for guideline inclusion. Furthermore, these models are seldom critically appraised for methodological quality. Despite the growing number of risk prediction models for POP in lung cancer, many issues remain unresolved.

Thus, this study systematically reviewed and evaluated research on POP risk prediction models for lung cancer patients, aiming to offer guidance and references for clinical practice and further research.

## 2 Methods

This systematic review and meta-analysis was conducted in accordance with the guidelines for systematic reviews of prediction model performance (16), and was reported following the TRIPOD-SRMA checklist (17). The study protocol was registered on PROSPERO (ID: CRD42024554224).

### 2.1 Search strategy

We conducted searches spanning several databases including China National Knowledge Infrastructure (CNKI), Wanfang Database, China Science and Technology Journal Database (VIP), SinoMed, PubMed, Embase, The Cochrane Library, Cumulative Index to Nursing and Allied Health Literature (CINAHL), and Web of Science. These databases were searched from their inception until July 20, 2026, using the following MeSH terms and keywords: “Lung Neoplasms”, “Postoperative pneumonia”, “Pneumonia”, “Pulmonary infection”, “Infection”, “Surgical Procedures, Operative”, “Risk prediction model”, “Prediction model”, “Risk factor”, “Predictor”, “Risk Score”, and “Predictive factors.” The detailed strategies used for the search can be found in the Supporting Information. Additionally, we increased the comprehensiveness of our literature review by examining the reference lists of the research articles we retrieved. The PICOTS framework assists in formulating the purpose of the review, defining the search strategy, and establishing inclusion and exclusion criteria for the study.

### 2.2 Inclusion and exclusion criteria

The inclusion criteria for studies included (1): studies involving patients diagnosed with lung cancer (2); studies employing an observational design (3); studies that reported a prediction model (4); studies where the outcome focused on POP. The exclusion criteria were (1): studies that did not report a predictive model (2); studies with outcomes limited to specific POP subgroups (3); studies not written in English or Chinese.

### 2.3 Study screening and data extraction

Two reviewers independently performed the study selection and data extraction. Any disagreements during the process were resolved through discussion, and a third reviewer was consulted to reach a final consensus when necessary. EndNote20 was used to identify and remove duplicate records. The titles and abstracts of retrieved studies were initially screened according to the predefined inclusion and exclusion criteria, followed by full-text assessment to determine the final eligible studies. Data extraction was conducted using a standardized form developed based on the CHARMS (Critical Appraisal and Data Extraction for Systematic Reviews of Prediction Modelling Studies) checklist (18). The extracted information included study characteristics, such as first author, publication year, country, study design, data source, study population, sample size, and outcome definition, as well as model characteristics, including modelling method, variable selection approach, handling of continuous variables, number and names of predictors, model development method, validation method, model performance measures, and model presentation format.

### 2.4 Quality assessment

Two reviewers also independently assessed the risk of bias and applicability of the included studies using the PROBAST checklist (19). The PROBAST checklist is designed to critically assess research involved in developing, validating, or updating prediction models for individualized predictions. It encompasses the domains of participant, predictor, outcome, and analysis, including 20 indicator questions that can be answered as “yes”, “probably yes”, “no”, “probably no” or “no information.” A domain is considered at high risk of bias if at least one indicator question within it is answered as “no” or “probably no.” A study is only considered to have a low overall risk of bias if all domains are assessed as low risk.

### 2.5 Data synthesis and statistical analysis

A meta-analysis of the AUC values from the validated models was conducted using Stata, version 17.0 software. The heterogeneity between studies was assessed using the I^2^ index and the Cochrane Q test. The I^2^ index quantifies heterogeneity, with values of 25%, 50%, and 75% indicating low, moderate, and high heterogeneity, respectively (20). Depending on the heterogeneity results, either a fixed or random effects model was applied. Additionally, Egger’s test was employed to detect publication bias, with P<0.05 suggesting publication bias (21). Sensitivity analysis was conducted to assess the robustness and reliability of the aggregated findings from the meta-analysis.

## 3 Results

### 3.1 Study selection

We retrieved a total of 3,044 related studies, and following the screening process, 20 studies were ultimately included. The literature screening process is illustrated in **Figure 1**.

**Figure 1:**
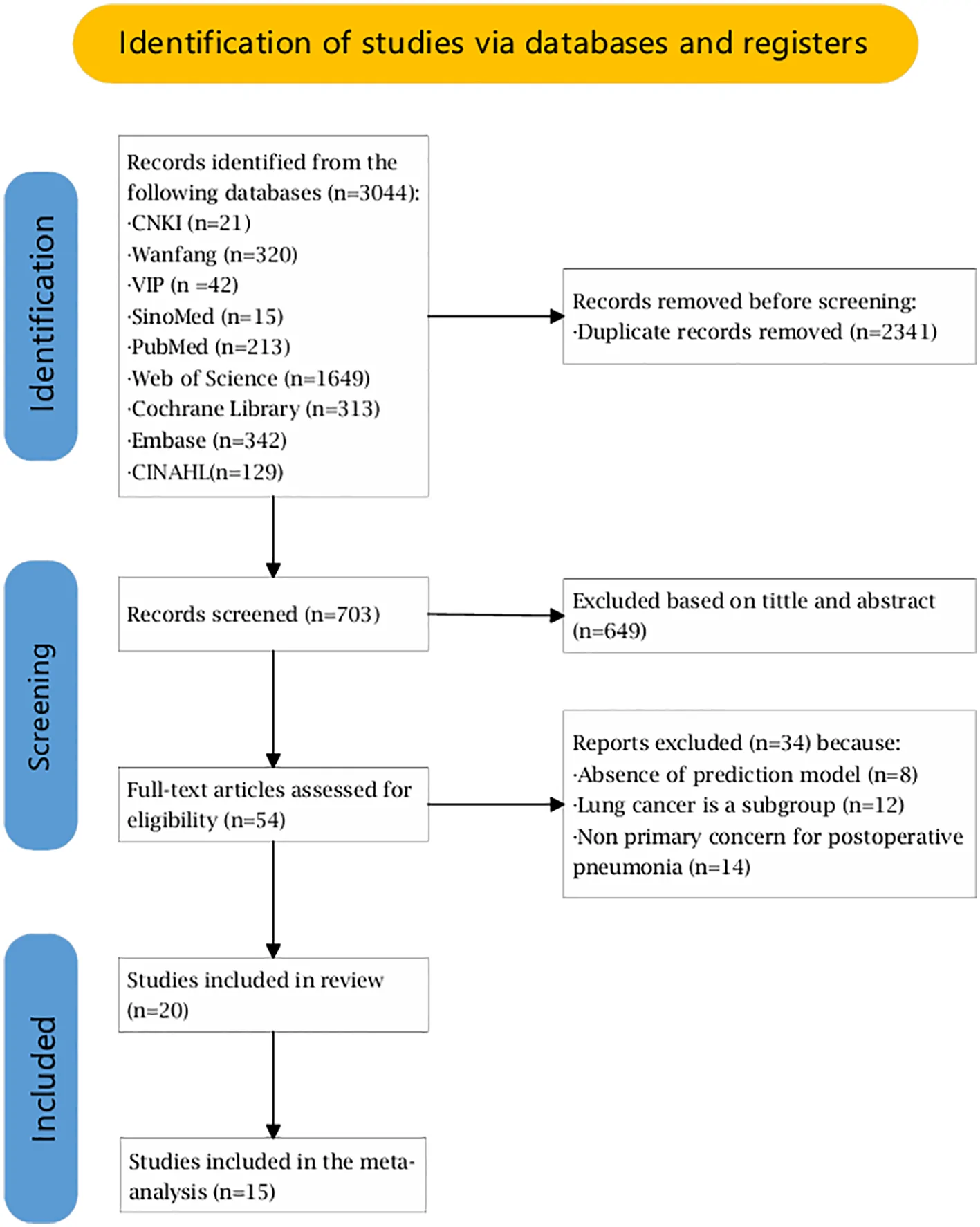
Literature screening process.

### 3.2 Study characteristics

The design and subject characteristics of the 20 studies included in this study are detailed in **Table 1**. These studies were published between 2019 and 2025, and all were conducted in China. Of the included studies, 17 were retrospective studies, while 2 were prospective cohort studies, including one multicenter study. In terms of the study subjects, 4 studies specifically mainly focused on lung cancer patients with adenocarcinoma or squamous cell carcinoma, 8 studies centered on non-small cell lung cancer patients, and the remaining 8 studies did not specify the type of lung cancer. 10 studies investigated lung cancer patients undergoing thoracoscopic surgery, 5 studies encompassed patients undergoing both thoracotomy and thoracoscopy, while the remaining 4 studies did not report the specific surgical approach. The sample sizes across these studies varied widely, ranging from 72 to 5,269 participants, involving a total of 18,558 lung cancer patients. The incidence of POP among these patients ranged from 1.7% to 27.8%.

**Table 1:** Overview of basic data of the included studies.

Author (year)	Country	Study design	Participants	Surgical approach	Main outcome	POP cases/sample size (%)
Ma et al. ^a^ (22)	China	Retrospective study	Non-small cell lung cancer patients	Thoracoscopy surgery	POP	226/2380 (9.50%)
Lu et al. ^a^ (23)	China	Retrospective study	Lung cancer patients	Thoracoscopy or thoracotomy surgery	POP	83/300 (27.7%)
Liu et al. ^a^ (24)	China	—	Non-small cell lung cancer patients	Thoracoscopy surgery	POP	39/204 (19.12%)
Su et al. (25)	China	Retrospective study	Mainly adenocarcinoma and squamous cell lung cancer patients	Thoracoscopy surgery	POP	64/457 (14.0%)
Zhang et al. (26)	China	Retrospective study	Non-small cell lung cancer patients	—	POP	131/1084 (12.1%)
Miao et al. (27)	China	Retrospective study	Lung cancer patients	Thoracoscopy surgery	POP	57/482 (14.8%)
Fu et al. ^a^ (28)	China	Retrospective study	Lung cancer patients	Thoracoscopy surgery	POP	35/126 (27.8%)
Ma et al. (29)	China	Retrospective cohort study	Non-small cell lung cancer patients	Thoracoscopy surgery	POP	39/872 (4.5%)
Jian et al. ^a^ (30)	China	Prospective cohort study	Non-small cell lung cancer patients	—	POP	130/730 (17.8%)
Zhang et al. (31)	China	Retrospective study	Lung cancer patients	Thoracoscopy surgery	POP	50/315 (15.9%)
Zhou et al. (32)	China	Retrospective study	Mainly adenocarcinoma and squamous cell lung cancer patients	Thoracoscopy or thoracotomy surgery	POP	92/5269 (1.7%)
Bian et al. (33)	China	Prospective cohort study	Mainly adenocarcinoma and squamous cell lung cancer patients	Thoracoscopy surgery	POP	196/1229 (16.0%)
Ding et al. (34)	China	Retrospective study	Non-small cell lung cancer patients	—	POP	27/224 (11.1%)
Jin et al. (35)	China	Retrospective study	Lung cancer patients	Thoracoscopy or thoracotomy surgery	POP	290/1252 (23.2%)
Li et al. ^a^ (36)	China	Retrospective study	Non-small cell lung cancer patients	Thoracoscopy or thoracotomy surgery	POP	136/745 (18.2%)
Cheng et al.^a^ (37)	China	Retrospective study	Lung cancer patients	—	POP	18/72 (25%)
Wang et al.^a^ (38)	China	Retrospective study	Lung cancer patients	Thoracoscopy surgery	POP	49/202 (24.3%)
Yu et al. (39)	China	Retrospective study	Mainly adenocarcinoma and squamous cell lung cancer patients	Thoracoscopy or thoracotomy surgery	POP	24/240 (10.0%)
Song et al. (40)	China	Retrospective study	Lung cancer patient	Thoracoscopy surgery	POP	162/1750 (9.3%)
Gao et al. ^a^ (41)	China	Retrospective study	Non-small cell lung cancer patients	Thoracoscopy surgery	POP	67/625 (10.7%)

^a^ Study was published in Chinese.

### 3.3 Model information

The model information from this study is detailed in **Table 2**. The included studies developed models predominantly using logistic regression, with machine-learning approaches used in two studies. These 20 studies collectively identified 40 final predictive factors. The most commonly used predictors across the models were age, operation time, smoking, diabetes mellitus, preoperative pulmonary function, Chronic obstructive pulmonary disease (COPD), and TNM stage, each featured in twelve, ten, nine, seven, six, five, and five studies respectively. Other factors such as the extent of excision, intraoperative blood loss, ASA grade, mechanical ventilation time, C-reactive protein, albumin, white blood cell count, BMI, drainage retention time, systemic immune-inflammation index (SII), procalcitonin, HMGB1, surgical approach, and many other predictive factors were each included in one to four of the studies. The reported AUC values ranged from 0.670 to 0.963.

**Table 2:** Overview of the information of the included prediction models.

	Model development	Model performance						
Author(year)	Variable selection	Model development method	AUC	Calibration method	Specificity/sensitivity	Validation method	Model presentation	Final predictors
Ma et al. ^a^ (22)	Lasso regression analysis	Lasso regression analysis, ANN, SVM, RF, XGB	0.941 (95% CI: 0.936-0.959) 0.936 (95% CI: 0.924-0.945) 0.881 (95% CI: 0.870-0.892) 0.923 (95% CI: 0.914-0.932) 0.962 (95% CI: 0.953-0.976)	—	0.915/0.832 0.903/0.814 0.929/0.881 0.932/0.855 0.937/0.896	Internal validation	Nomogram	Daily cigarette consumption, Diabetes, Preoperative diffusing capacity, Maximal tumor diameter, 24-hour postoperative chest drainage volume, ONS, Postoperative urinary catheterization, Intraoperative pleural adhesion severity
Lu et al. ^a^ (23)	Univariate analysis	Multivariable logistic regression analysis	0.897 (95% CI: 0.849-0.945)	Calibration curve, Hosmer-Lemeshow Test:*χ2* = 6.557, *P* = 0.585	—	Internal validation	Nomogram and Formula	Age, Surgical approach, Use of dexmedetomidine, Operative time, Postoperative invasive procedure
Liu et al. ^a^ (24)	Univariate analysis	Multivariable logistic regression analysis	0.902(95% CI: 0.851~0.953)	Calibration curve, Hosmer-Lemeshow Test:*χ2* = 0.769, *P* = 0.513	0.921/0.683	Internal validation	Nomogram	Age, TNM clinical stage III, mechanical ventilation time ≥ 12h, elevated C-reactive protein levels
Su et al. (25)	Univariate analysis	Multivariable logistic regression analysis	0.839 (95%CI:0.782-0.890)	Calibration curve, Hosmer-Lemeshow Test: *χ2* = 1.52, *P* = 0.175	0.521/0.935	Internal validation	Nomogram	Ki67 Proliferation Index, Age, Preoperative pulmonary function, BMI
Zhang et al. (26)	Univariate analysis	Multivariable logistic regression analysis, SVM, XGB	0.935(95%CI:0.910-0.960) 0.945 (95%CI:0.922-0.968) 0.963 (95%CI:0.944-0.982)	Calibration curve	0.855/0.909 0.863/0.930 0.908/0.906	Internal validation	Nomogram	Tumor stage, Diabetes history, COPD, Operation duration, Mechanical ventilation duration, Age, C-reactive protein; Procalcitonin, High-mobility group box 1, Interleukin-6, Neutrophil-to-lymphocyte ratio, Platelet-to-lymphocyte ratio, Systemic immune-inflammation index
Miao et al. (27)	Univariate analysis	Multivariable logistic regression analysis	0.790 (95% CI: 0.71–0.86)	—	0.811/0.786	—	Formula	SII, Prealbumin
Fu et al. ^a^ (28)	Univariate analysis	Multivariable logistic regression analysis	0.824 (95% CI: 0.753-0.895)	Calibration curve, Hosmer-Lemeshow Test:*χ2* = 8.448, *P* =0.391	0.731/0.791	Internal validation	Nomogram	Smoking. COPD, Diabetes, Age, Serum albumin., WBC
Ma et al. (29)	Univariate analysis	Multivariable logistic regression analysis	0.763(95%CI0.683–0.842)	Calibration curve	—/—	Internal validation	Nomogram	Surgical site, Tumor diameter, Thoracic drainage retention time, Pan-immune-inflammation value
Jian et al. ^a^ (30)	Univariate analysis	Binary logistic regression model	0.921(95%CI:0.818-0.984)	Calibration curve	0.918/0.973	Internal validation and External validation	Nomogram	Age, COPD, Mechanical ventilation time, CRP, PCT, SAA, HMGB1, sCD14, CD4+/CD8+
Zhang et al. ^a^ (31)	Univariate analysis	Binary logistic regression model	0.907	Calibration curve	—	Internal validation	Nomogram	Age (≥62.5years), Smoking index≥100, PEF ≤ 72.1 ml/s. TNM staging III/IV, Operation time≥188.5 min
Zhou et al. (32)	Univariate analysis	Multivariable logistic regression analysis	0.821(95%CI:0.783-0.859)	Calibration curve. Hosmer-Lemeshow Test:*χ2* = 6.660, *P* =0.580	—	Internal validation	Nomogram	Age, pFEV1%, pDLCO%, Tuberculosis history, Surgery duration
Bian et al. (33)	Univariate analysis	Binary logistic regression model	0.851 (95%CI: 0.821–0.881)	Calibration curve	0.801/0.744	Internal validation	Nomogram	Age, High BMI, Smoking, Poor physical condition, Respiratory diseases, Diabetes mellitus, Neurological diseases
Ding et al. (34)	Univariate analysis	Multivariable logistic regression analysis	0.894	Calibration curve	0.912/0.786	Internal validation	Nomogram and Formula	Age, DM, TNM staging, Chemotherapy regimen, Chemotherapy cycle, Post-chemotherapy albumin (g/L), Pre-chemotherapy KPS, Operation time
Jin et al. (35)	Univariate analysis	Multivariable logistic regression analysis	0.732(95%CI:0.676-0.801)	Calibration curve, Hosmer-Lemeshow Test: *χ2* = 1.953, *P* = 0.377	—/—	Internal validation and External validation	Nomogram	Smoking, Diabetes mellitus, history of preoperative chemotherapy, Thoracotomy, ASA grade, Surgery time
Li et al. ^a^ (36)	Univariate analysis	Multivariable logistic regression analysis	0.747	Calibration curve, Hosmer-Lemeshow Test: *P* = 0.698	—/—	Internal validation	Nomogram and Formula	Smoking index≥400, DM, Postoperative obvious pain, Chest bottle indwelling time>4d
Cheng et al. ^a^ (37)	Univariate analysis	Multivariable logistic regression analysis	0.816	Hosmer-Lemeshow Test: *χ2* = 8.372, *P* = 0.14	—/—	Internal validation	Nomogram	Age, smoking, FEV1, Operation time, Extent of excision, intraoperative blood loss
Wang et al. ^a^ (38)	Univariate analysis	Multivariable logistic regression analysis	0.852(95%CI:0.775-0.929)	Calibration curve, Hosmer-Lemeshow Test: *χ2* = 4.059, *P* = 0.541	0.737/0.844	Internal validation and External validation	Nomogram and Formula	Operation time>175min, ASA >3points, Combined chronic lung disease
Yu et al. (39)	Univariate analysis	Binary logistic regression model	0.827(95%CI:0.734–0.919)	—	0.708/0.824	Internal validation	Formula	Heart disease, Need for sputum suctioning, BMI, Postoperative WBC count
Song et al. (40)	Univariate analysis	Multivariable logistic regression analysis	0.67	Calibration curve	0.633/0.412	Internal validation and External validation	Formula	Sex, Smoking, history of COPD, Surgery duration, Leukocyte count, Intraoperative injection of colloid, Intraoperative injection of hormone
Gao et al. ^a^ (41)	Univariate analysis	Multivariable logistic regression analysis	0.909(95%CI:0.872-0.946)	Hosmer-Lemeshow Test: *χ2* = 4.322, *P* = 0.872	—/—	Internal validation	Nomogram and Formula	Age, Preoperative FEV1%<80%, pathological stage, Extent of excision, Smoking index, Operation time, Intraoperative blood loss

^a^ Study was published in Chinese.’-’, not reported; ASA, American Society of Anesthesiologists; ONS, Perioperative oral nutritional supplementation; FEV1, Forced expiratory volume in one second; COPD, chronic obstructive pulmonary disease; CRP, creactive protein; PCT, procalcitonin; SAA, serum amyloid A;HMGB1, high mobility group box 1; sCD14,soluble CD14; PEF, peak expiratory flow; KPS, karn of sky performance score; SII, the systemic immune-inflammation index; DM, diabetes mellitus; TNM, tumor node metastasis; pFEV1%, predicted forced expiratory volume in 1 s; pDLCO%, predicted diffusing capacity of the lungs for carbon monoxide; BMI, Body Mass Index; WBC, white blood cell. AUC, area under the curve. We considered AUC=0.5–0.7 as poor discrimination, 0.7–0.8 as moderate discrimination, 0.8–0.9 as good discrimination, and 0.9–1.0 as excellent discrimination. 3.4 Risk of bias and applicability assessment.

This study summarized the risk of bias and applicability concerns in the included studies, as detailed in **Table 3**. All studies evaluated were found to have a high risk of bias, indicating methodological issues in their development or validation processes.

**Table 3:** PROBAST results of the included studies.

	ROB				Applicability			Overall		
Author (year)	Participants	Predictors	Outcome	Analysis	Participants	Predictors	Outcome	ROB	Applicability	
Ma et al. ^a^ (22)	–	+	–	–	–	+	+	–	–	
Lu et al. ^a^ (23)	–	+	+	–	+	+	+	–	+	
Liu et al. ^a^ (24)	?	+	–	–	–	+	+	–	–	
Su et al. (25)	–	+	+	–	–	+	+	–	–	
Zhang et al. (26)	–	+	+	+	–	+	+	–	–	
Miao et al. (27)	–	+	+	–	–	+	+	–	–	
Fu et al. ^a^ (28)	–	+	?	–	–	?	+	–	–	
Ma et al. (29)	–	?	+	–	–	–	+	–	–	
Jian et al. ^a^ (30)	+	?	+	+	–	–	+	–	–	
Zhang et al. ^a^ (31)	–	+	+	–	–	+	+	–	–	
Zhou et al. (32)	–	–	–	–	+	+	+	–	+	
Bian et al. (33)	+	–	–	–	–	+	+	–	–	
Ding et al. (34)	–	?	+	–	–	–	+	–	–	
Jin et al. (35)	–	+	+	–	+	+	+	–	+	
Li et al. ^a^ (36)	–	+	–	–	–	+	–	–	–	
Cheng et al. ^a^ (37)	–	+	+	–	+	+	+	–	+	
Wang et al. ^a^ (38)	–	+	+	–	–	+	+	–	–	
Yu et al. (39)	–	–	–	–	–	+	+	–	–	
Song et al. (40)	–	–	–	–	–	+	+	–	–	
Gao et al. ^a^ (41)	–	+	+	–	–	+	+	–	–	

PROBAST, Prediction model Risk of Bias Assessment Tool; ROB, risk of bias.

^a^ Study was published in Chinese; + indicates low ROB/low concern regarding applicability; - indicates high ROB/high concern regarding application;? indicates unclear ROB/unclear concern regarding applicability.

In the participant domain, 17 studies were deemed to have a high risk of bias, and 1 study was deemed to indicate an unclear risk of bias. primarily due to the use of inappropriate data sources (22–29, 31, 32, 34–41). In the predictor domain, 4 studies exhibited an unclear risk of bias due to the similarity in definitions of predictive factors and outcome indicators (32, 33, 39, 40); 3 studies showed an unclear risk because they did not provide information on all predictive factors at the time of model use (29, 30, 34). In the outcome domain, 8 studies were identified as having a high risk of bias: 4 studies failed to exclude information on predictive factors in defining outcomes (32, 33, 39, 40), and 4 studies did not ensure an appropriate time interval between predictor assessment and outcome determination (22, 24, 28, 36). In the analysis domain, 18 studies displayed a high risk of bias. Of these, 11 studies had insufficient sample sizes, failing to meet the guideline of more than 20 ‘events per variable’ (EPV) (23–25, 27, 28, 31, 34, 36–39); 4 studies converted continuous variables into categorical variables, either partially or fully (32–34, 39); 1 study excluded some enrolled participants from the analysis, and the handling of missing data was inadequate (40); 8 studies did not refrain from selecting variables based on univariate analysis (29, 31–33, 36–38, 41); 8 studies did not thoroughly evaluate the predictive performance of their models (29, 31, 32, 35–37, 39, 41); 3 studies failed to address issues like overfitting and underfitting of model performance (22, 27, 39); 2 studies did not disclose the coefficients of predictors in the multivariate regression model (36, 37).

Regarding the assessment of applicability risk, 16 studies were assessed as having a high risk and 4 as having a low risk. In the participant domain, due to the inclusion of participants limited to specific subgroups of lung cancer patients, 16 studies were identified with high applicability risk (22, 24–31, 33, 34, 36, 38–41); The main reasons are as follows: 6 studies focused on particular types of lung cancer (26, 29, 30, 34, 36, 41), 2 studies targeting elderly lung cancer patients (39, 40), and 11 studies involving lung cancer patients undergoing thoracoscopic surgery (22, 24, 25, 27–29, 31, 33, 38, 40, 41). In the predictor domain, 4 studies were deemed to have a high risk of applicability due to concerns about the timing of predictor measurements (28–30, 34). In the outcome domain, 1 study presented a high applicability risk because the evaluation time interval for postoperative outcomes was too long (36).

### 3.5 Meta-analysis of validation models included in the review

Only 21 prediction models across 15 studies met the eligibility criteria for inclusion in this study.

The pooled AUC, calculated using a random effects model, yielded a value of 0.89 (95% CI: 0.87-0.91) as depicted in **Figure 2**. The I^2^ value was 92.8% (p<0.001), indicating a high degree of heterogeneity among the studies. Additionally, Egger’s test yielded a value of 2.243 (p=0.016), suggesting significant publication bias.

**Figure 2:**
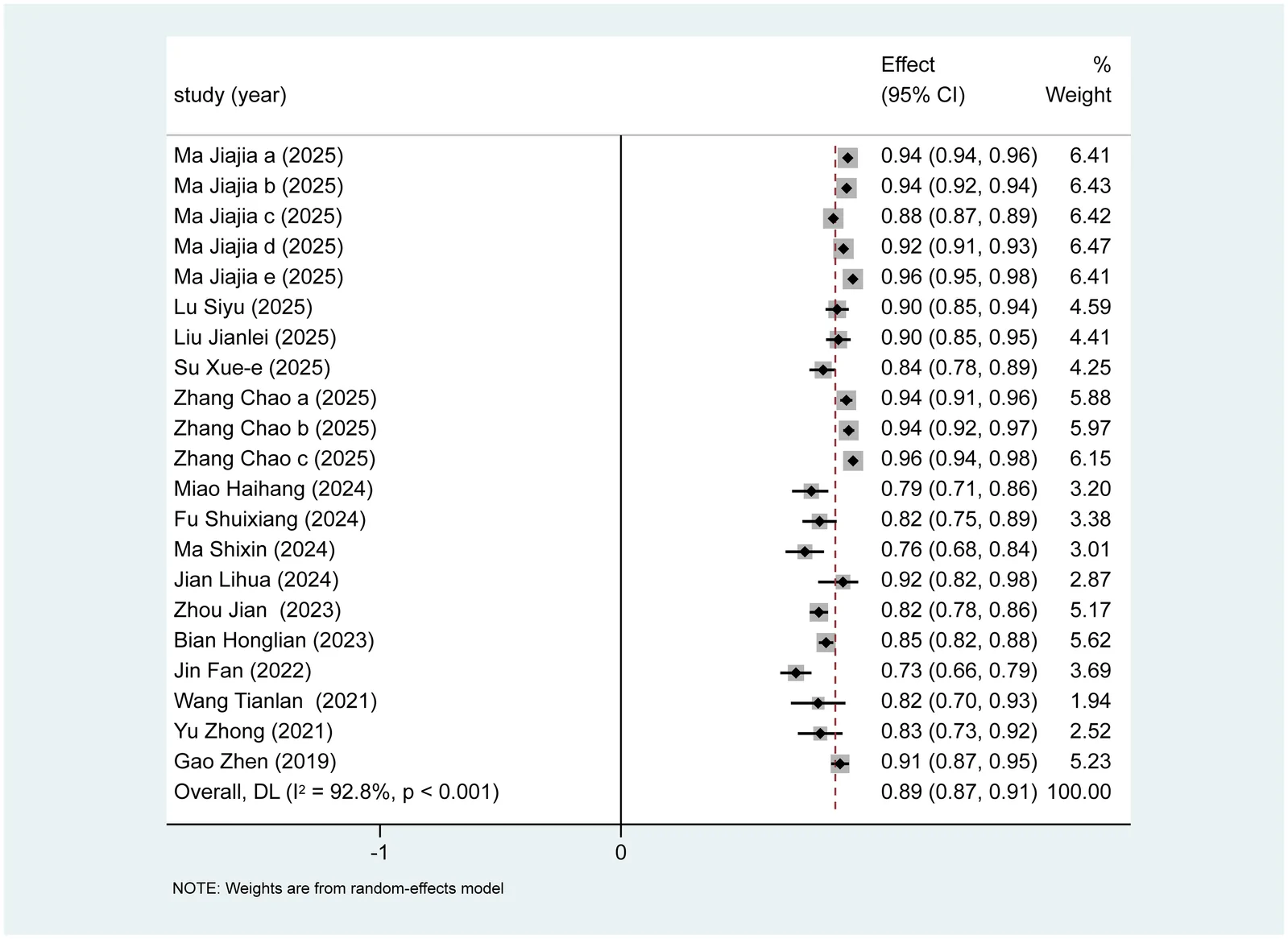
Forest plot of the random effects meta-analysis of pooled AUC of 21 validation models.

### 3.6 The sensitivity analysis of validation models included in the review

The sensitivity analysis results indicated that after excluding each model one by one, the combined effect size did not change significantly, which shows that the research results have strong robustness and reliability (**Figure 3**).

**Figure 3:**
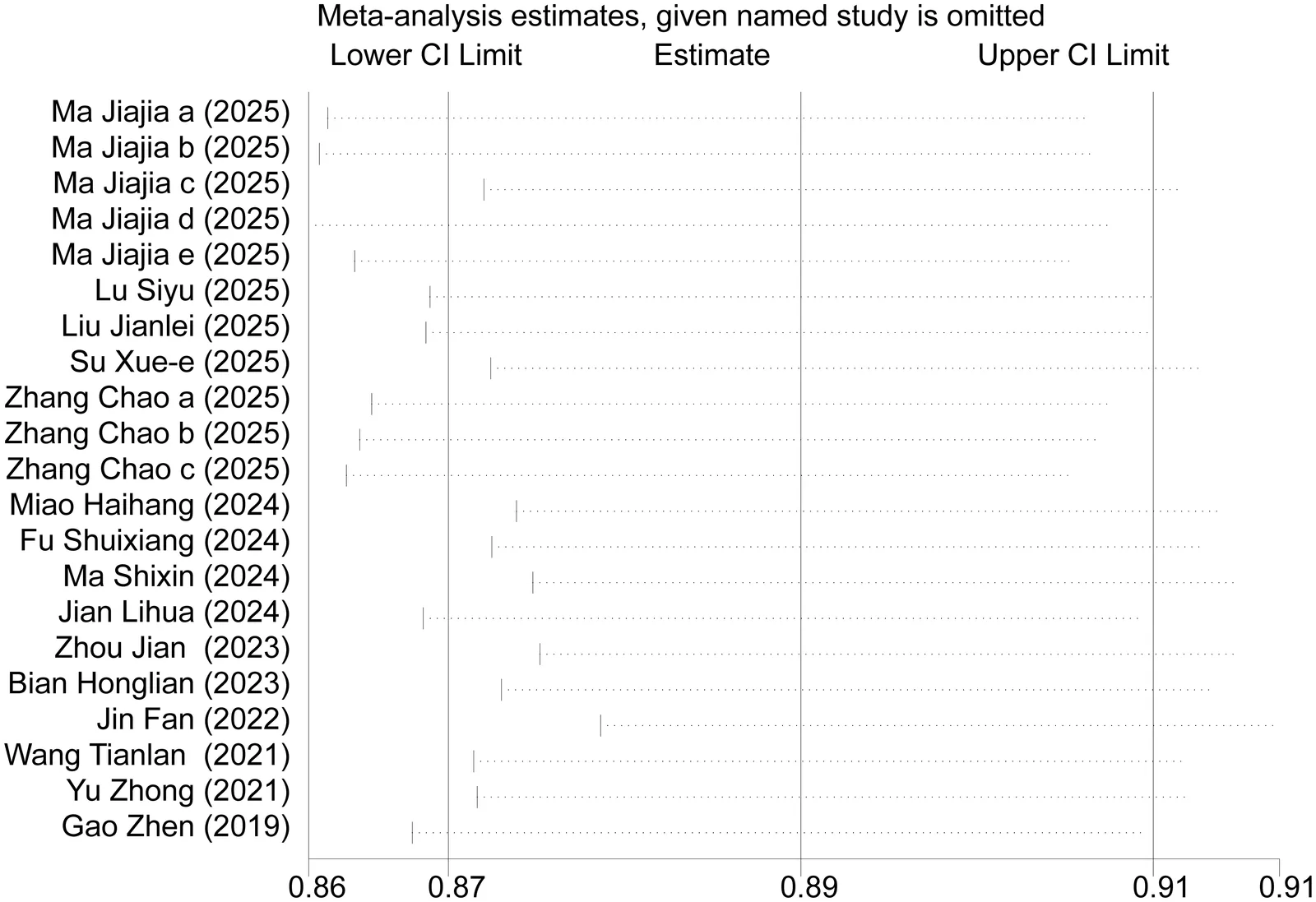
Sensitivity analysis results.

### 3.7 The subgroup analysis of validation models included in the review

A random-effects model was applied, and subgroup analysis was performed across the 21 prediction models identified from the 15 included studies according to whether external validation was conducted. We excluded one model from the subgroup analysis because it did not report its validation method. The remaining 20 predictive models were included in the subgroup analysis. As shown in **Figure 4**, considerable heterogeneity was observed in the internal validation subgroup (I² = 92.9%, p < 0.001).

**Figure 4:**
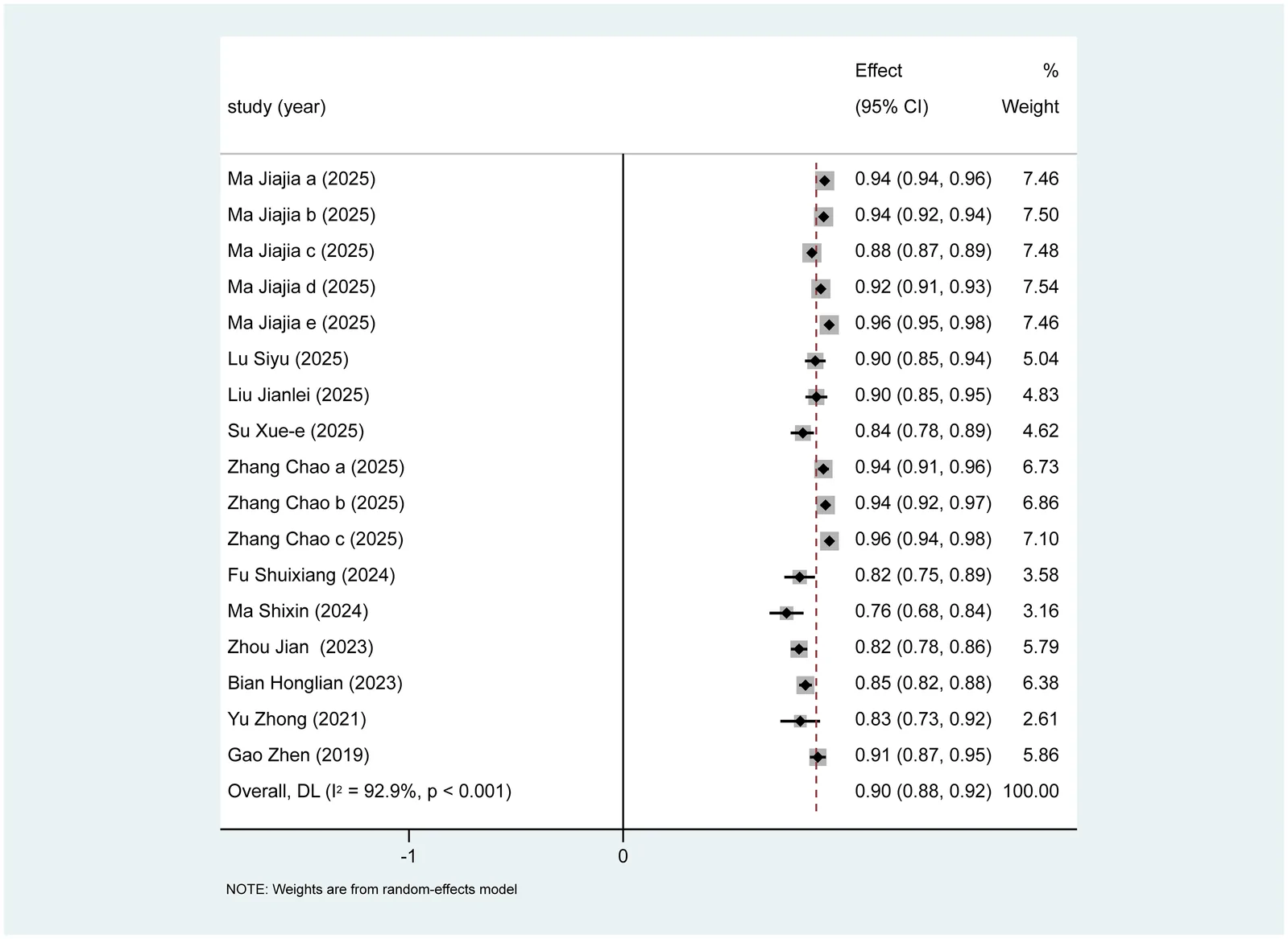
Subgroup analysis of prediction models with internal validation.

As shown in **Figure 5**, the subgroup involving both internal and external validation demonstrated substantial heterogeneity (I² = 85.0%, p < 0.001).

**Figure 5:**
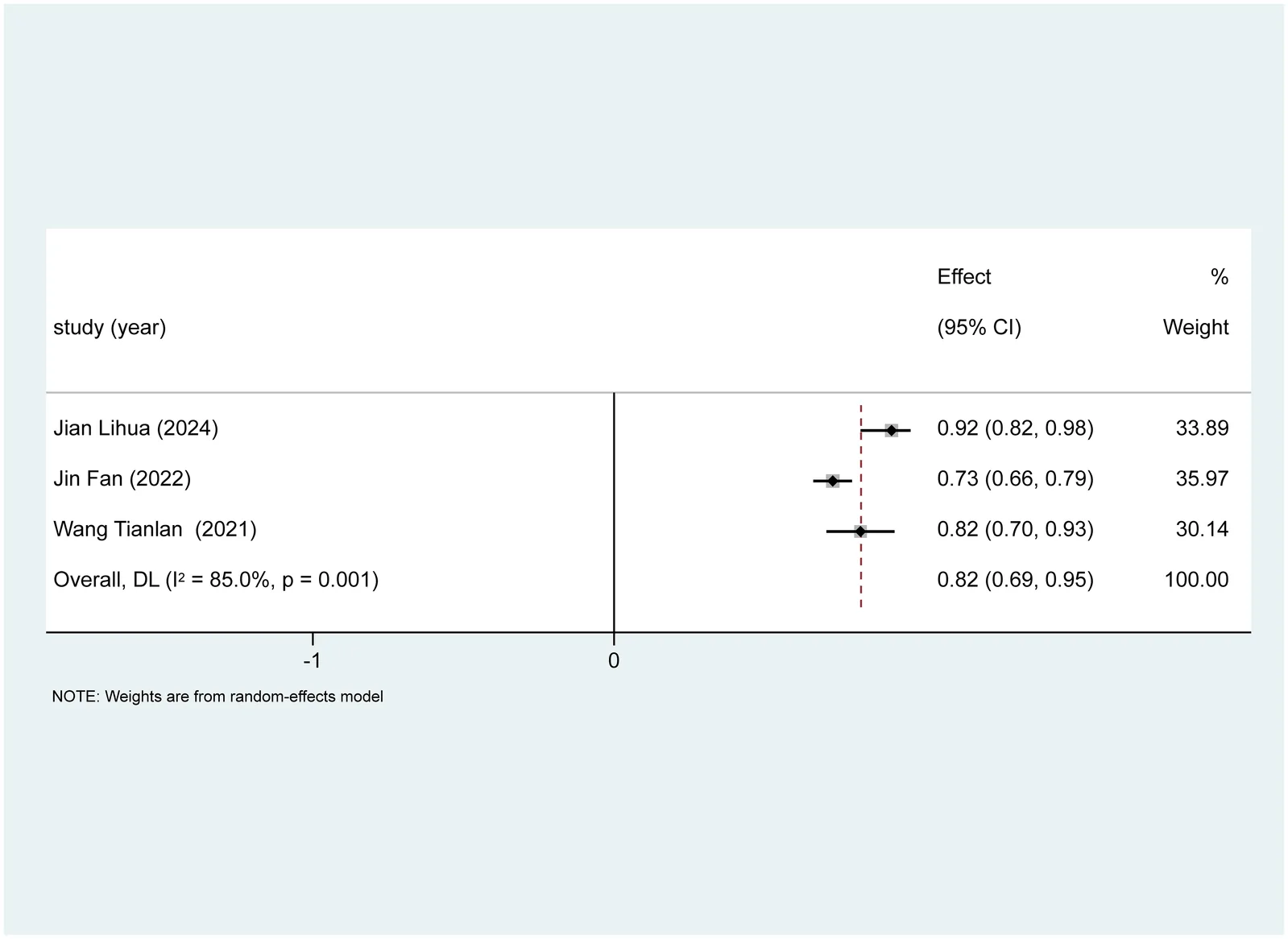
Subgroup analysis of prediction models with internal and external validation.

## 4 Discussion

Multiple studies have demonstrated that lung cancer patients are more susceptible to postoperative pulmonary complications (POP) than patients with other cancers (42, 43). POP can be mitigated through early identification and prediction, followed by targeted interventions (11, 12). Developing a model to predict the risk of POP in lung cancer patients can facilitate the creation of risk reduction strategies and the allocation of resources for postoperative care (44, 45). Predictive models are essential for early detection and intervention in assessing the potential risk of POP in lung cancer patients. This review highlights an increasing number of risk prediction models for POP in lung cancer patients, although these models primarily utilize data from Chinese patients. We evaluated 26 predictive models from 20 studies that demonstrated moderate to excellent predictive performance, with reported AUC values ranging from 0.670 to 0.963. However, all studies were assessed as having a high risk of bias according to the PROBAST, which limits the practical utility of these prediction models. The pooled AUC value of the 21 validated models included in the meta-analysis was 0.89 (95% CI: 0.87-0.91). Nevertheless, the heterogeneity among the models was substantial, possibly due to differences in methodology, predictors, and populations of the different models. The literature included in this study mostly used internal validation to develop models, and we only merged AUC without merging calibration and other data, which may inflate the AUC. Future studies should therefore prioritize developing new better-quality models to advance in this field.

Valuable insights can be gained from the development process of these models. Notably, two models in this study specifically targeted elderly lung cancer patients. While POP is a common complication after lung surgery (46) and more prevalent among the elderly, it can affect patients of any age, necessitating screening across all age groups. Furthermore, Bian’s (33) study employed a prospective cohort design with a large sample size but lacked external validation, failing to test the transferability and generalization of the model (47). In contrast, the studies by (22, 30, 40), despite having large sample sizes and conducting both internal and external validations, used retrospective designs, thereby introducing a high risk of bias. The definition of predictive factors and outcome indicators in Song et al. ‘s (40) study was similar, posing a risk of bias in the participant, predictor, and outcome domains. However, their research merits recognition for incorporating variables such as smoking and COPD score, based on previous studies, thus avoiding variable selection based solely on univariate analysis. Most of the models in this study were built using logistic regression. Many studies encounter challenges related to sample size, handling of continuous variables, selection of predictive variables, and model evaluation. Research indicates that machine learning methods often yield higher accuracy than traditional logistic regression. Some of these issues might be addressed by integrating machine learning techniques into model development. Only two studies Ma et al. (22), and Zhang et al. (26) have applied machine learning to construct models, but due to the lack of external validation, the repeatability and generalization ability of the models are still unclear. Although all models show moderate to excellent performance, the risk of bias remains a significant concern. Future researchers should aim to rectify these shortcomings and select appropriate model development methodologies based on the specific context.

The predictive models discussed in this review hold significant clinical relevance. First, frequently identified predictive factors provide valuable insights for nursing practice and future research. Age is widely recognized as a risk factor for postoperative pulmonary complications (POP) in lung cancer patients (42, 46, 48), with the incidence of POP increasing with age (8). Therefore, medical staff should be particularly alert to the risk of POP in elderly lung cancer patients. Additionally, prolonged surgical times are associated with an increased risk of POP (49, 50). Longer surgeries extend the duration of mechanical ventilation and anesthesia, potentially reducing pulmonary surfactant levels and leading to postoperative pulmonary infections (29, 51). However, this does not imply that surgical durations should be unnecessarily shortened. Instead, strategies that reduce operating times without compromising safety are essential for surgeons and healthcare systems (52). Smoking is another well-documented risk factor for POP (2, 53, 54), as it impairs the mucociliary escalator’s ability to clear foreign bodies and pathogens from the airways (55). Nonetheless, there is variability in how smoking status is quantified in different studies. Some research highlights the benefits of quitting smoking before surgery, regardless of the patient’s previous smoking history (56). Diabetes has also been linked to an increased risk of POP following surgery (57, 58), with potential contributing factors including compromised pulmonary immunity, diminished lung function, and reduced cardiorespiratory fitness, which heighten susceptibility to infections (59). Forced expiratory volume in one second FEV1 is also a crucial predictive factor for POP (48, 60), though there is variation in the specific FEV1 values considered in different studies. More research is needed to validate these findings. Research has shown that previous pneumonia and Postoperative lymphopenia are influencing factors of POP in lung cancer patients (6, 8). However, the predictive model included in this study did not cover these factors. In the future, further research can be conducted to determine whether these influencing factors have predictive effects on POP in lung cancer patients. Lastly, variations in study populations and methodologies among different institutions can lead to inconsistent results and increase the risk of bias. Thus, while there is an urgent need for the clinical implementation of POP prediction models for lung cancer patients, more clinical trials are required to confirm the efficacy of current models in reducing POP incidence and to potentially broaden their applicability to a wider patient population.

This systematic review has several limitations. First, all the studies included were conducted in China, which may necessitate adjustments when applying these models in different countries. Second, our meta-analysis incorporated only 21 models, potentially leading to significant inter-study heterogeneity due to variations in reporting transparency, surgical techniques, and research methodologies among the studies. Third, the models included in this study may not cover all factors influencing POP in lung cancer patients, which may affect the predictive performance of the model. Finally, this study was restricted to articles published in English and Chinese, potentially overlooking other valuable risk assessment tools published in different languages.

## 5 Conclusion

This systematic evaluation encompassed 20 studies featuring 26 models and found a pooled AUC of 0.89 (95% CI: 0.87-0.91) for 21 validated models, suggesting reasonable discrimination; however, their clinical applicability is limited by a high risk of bias, which may lead to overestimation of discrimination. However, all included studies were assessed as having a high risk of bias according to PROBAST, and 16 studies raised concerns regarding their applicability. Consequently, the current prediction models for POP in lung cancer patients fall short of meeting PROBAST standards. Therefore, researchers should adhere to the reporting guidelines specified in the TRIPOD statement and familiarize themselves with the PROBAST checklist to improve the quality of future research. Future studies on POP risk prediction models for lung cancer patients should focus on developing new models that incorporate larger sample sizes, more rigorous research designs, and multicenter external validations.

## Data Availability

The raw data supporting the conclusions of this article will be made available by the authors, without undue reservation.
